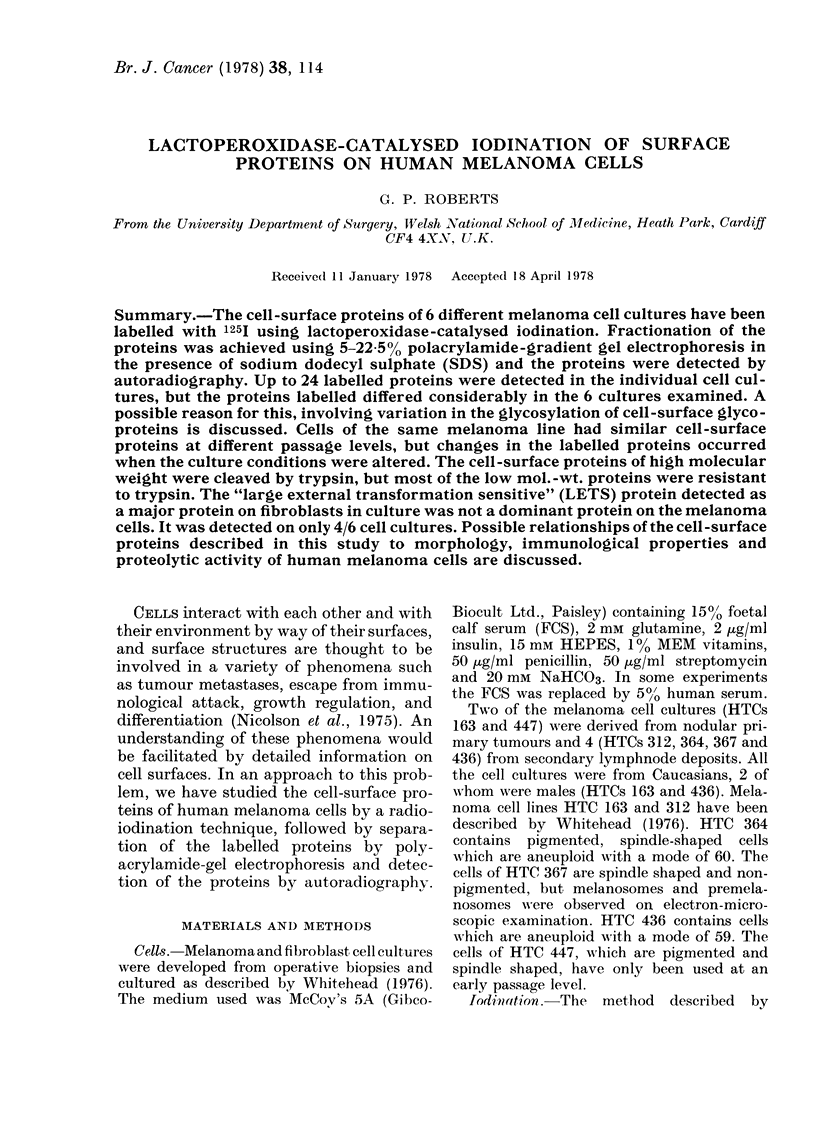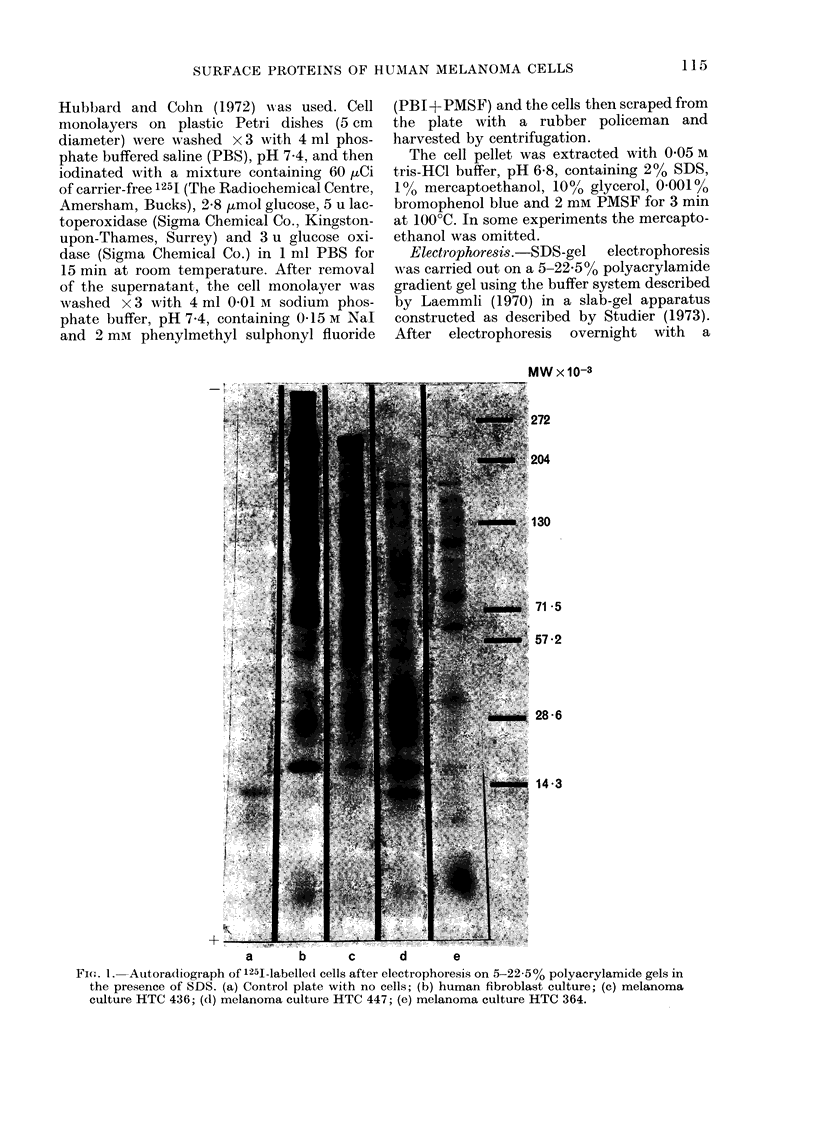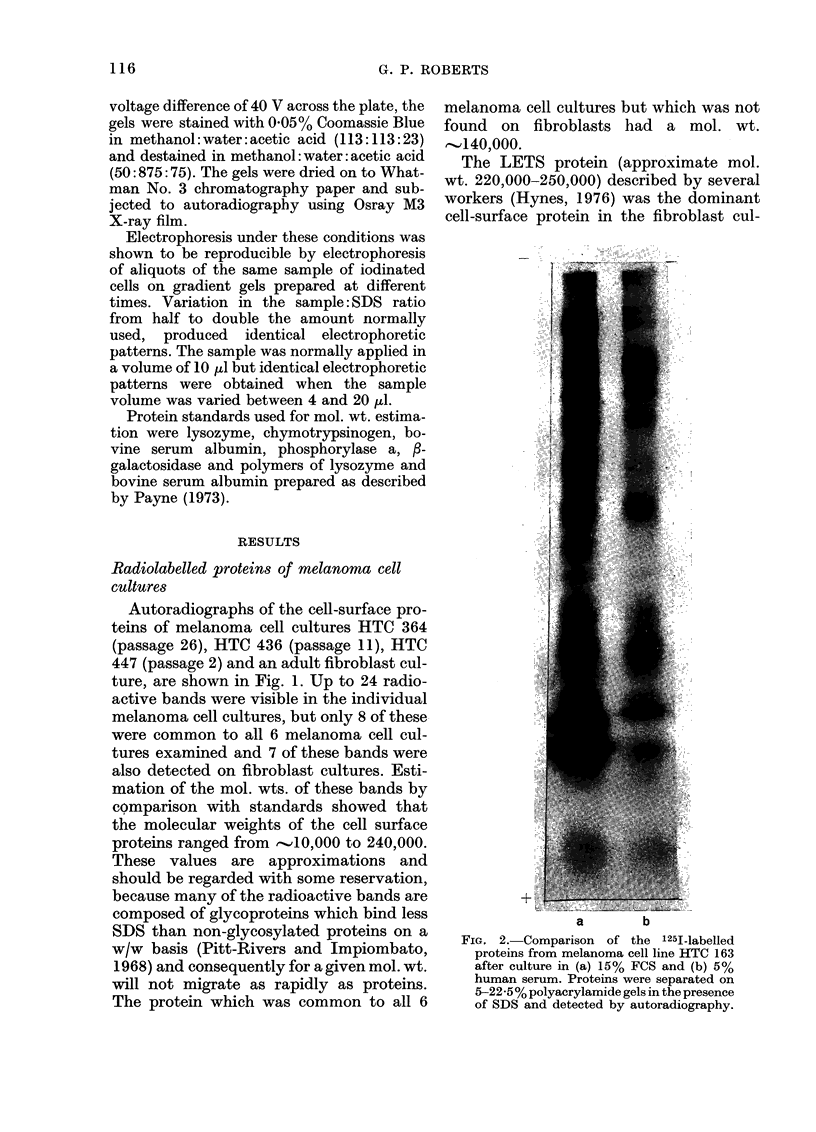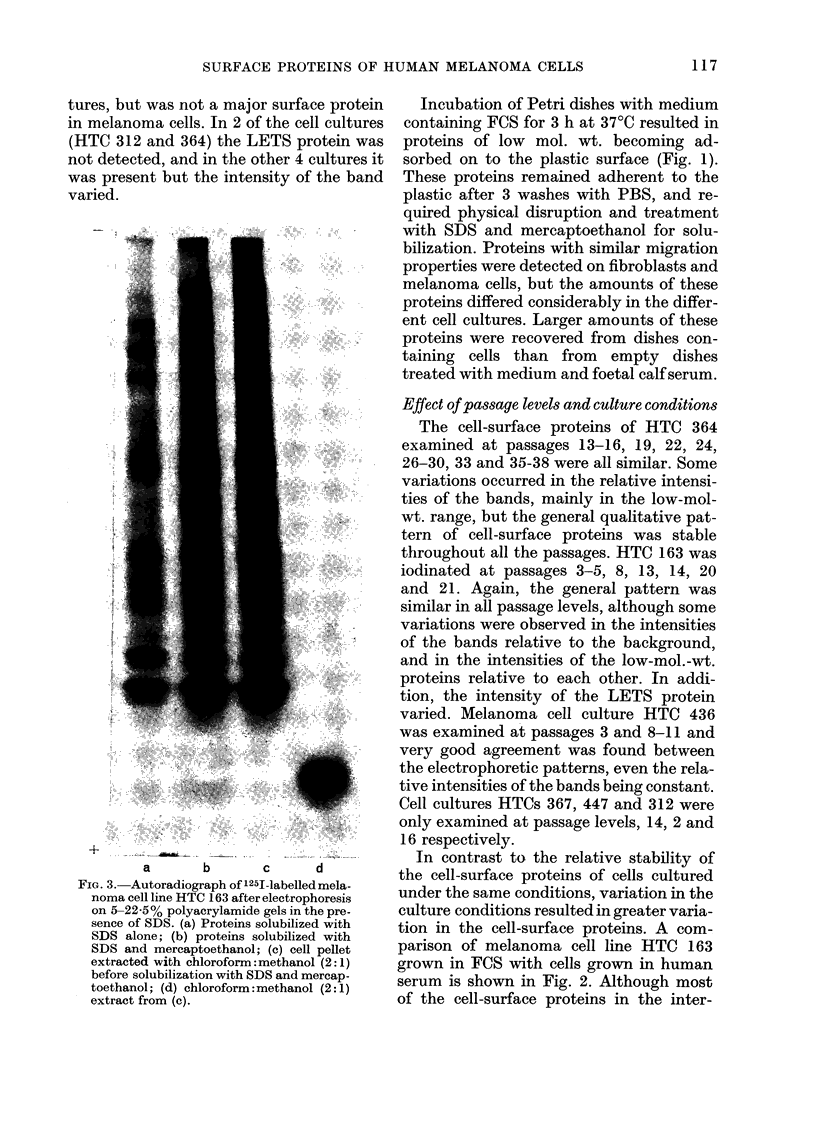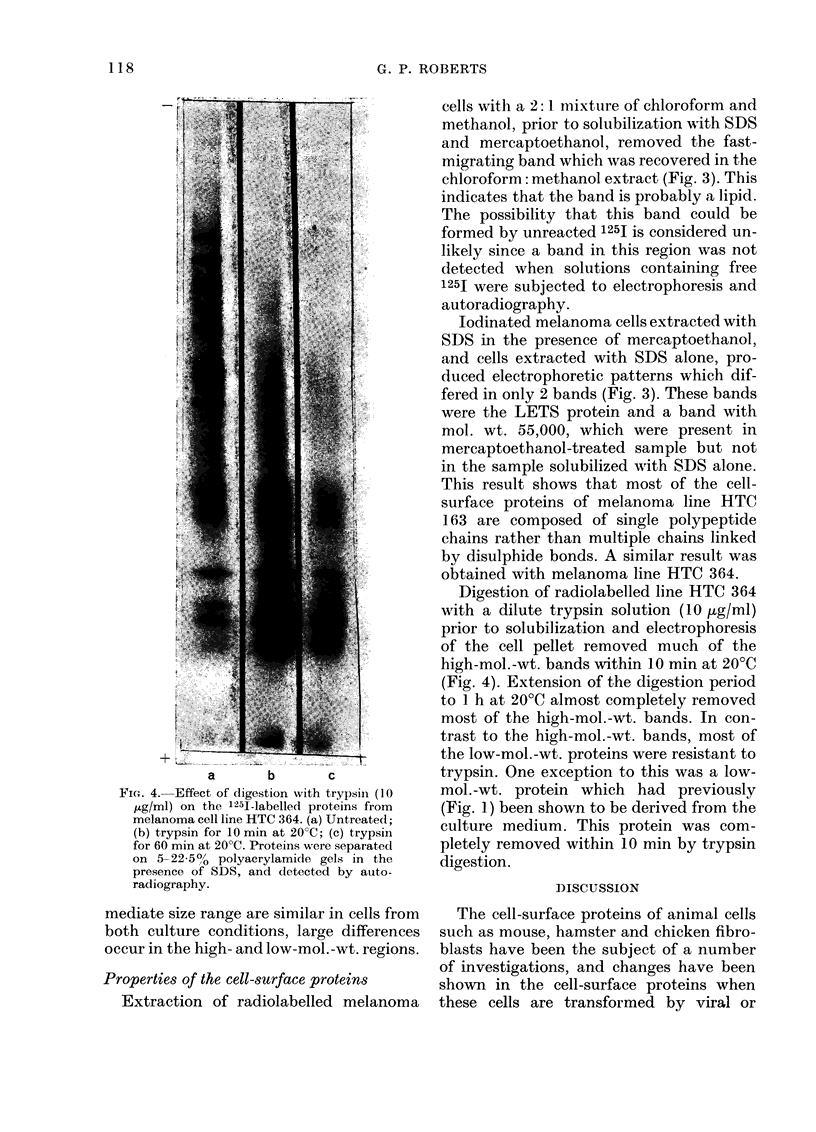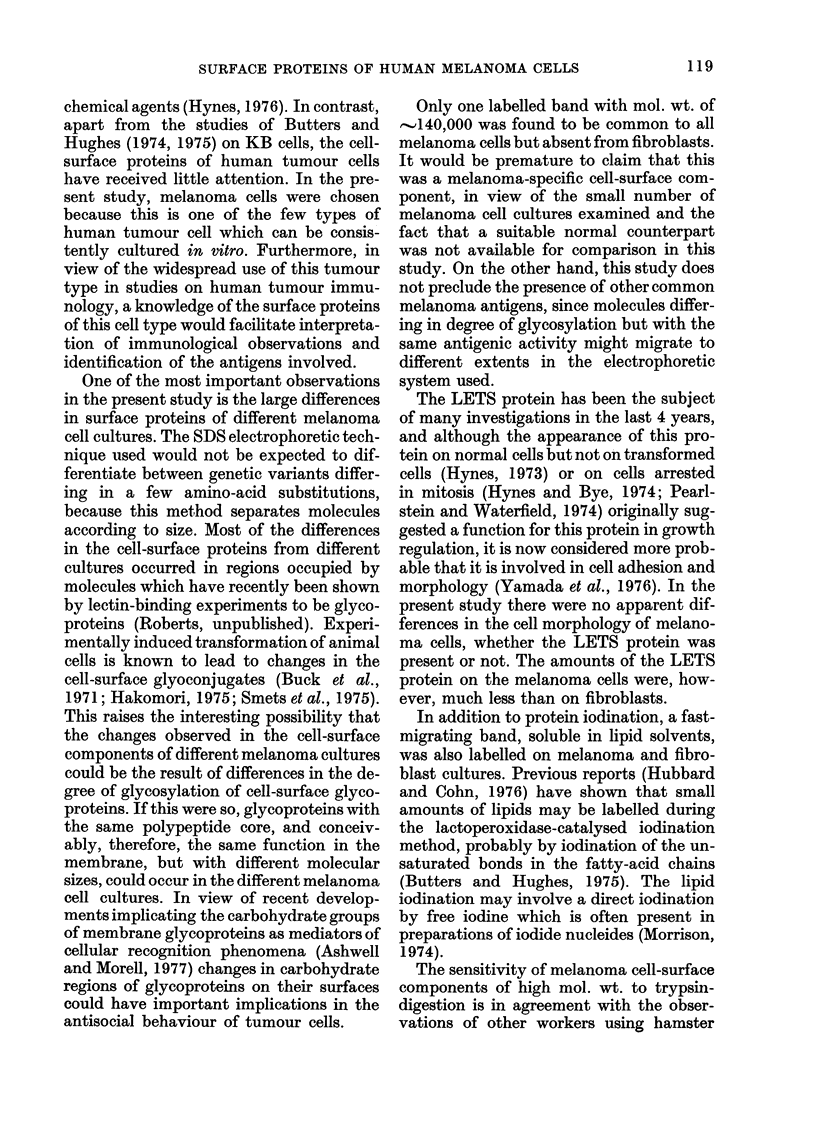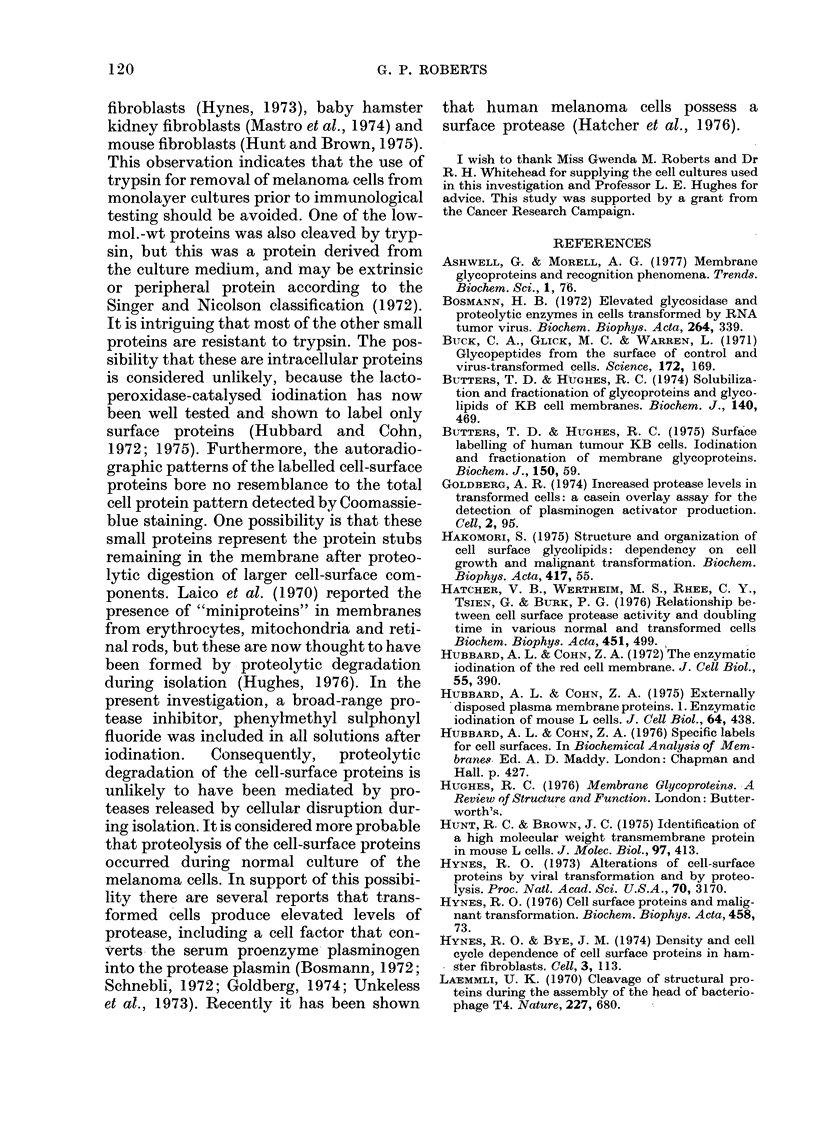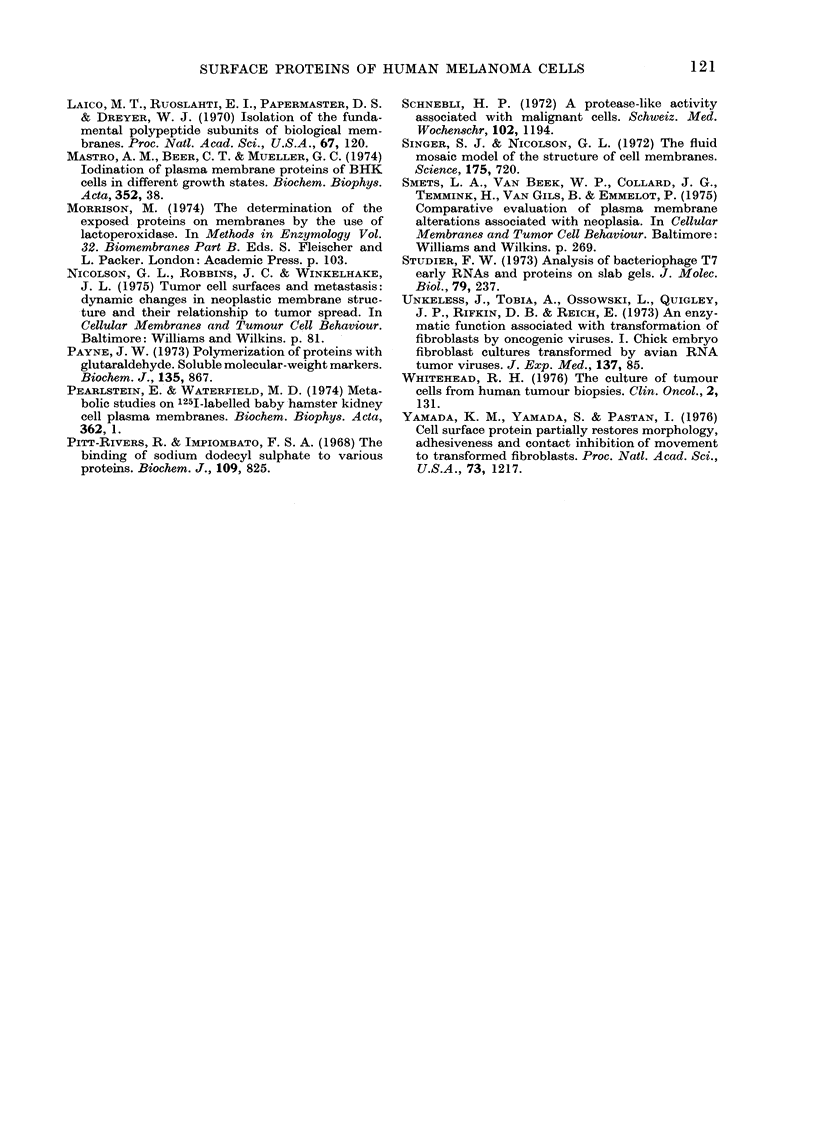# Lactoperoxidase-catalysed iodination of surface proteins on human melanoma cells.

**DOI:** 10.1038/bjc.1978.170

**Published:** 1978-07

**Authors:** G. P. Roberts

## Abstract

**Images:**


*Br. J. Cancer* (1978) **38**, 114

### LACTOPEROXIDASE-CATALYSED IODINATION OF SURFACE PROTEINS ON HUMAN MELANOMA CELLS

G. P. ROBERTS

*From the University Department of Surgery, Welsh National School of Medicine, Heath Park, Cardiff CF4 4XN, U.K.*

Received 11 January 1978 Accepted 18 April 1978

**Summary.—The cell-surface proteins of 6 different melanoma cell cultures have been labelled with $^{125}$I using lactoperoxidase-catalysed iodination. Fractionation of the proteins was achieved using 5–22·5% polacrylamide-gradient gel electrophoresis in the presence of sodium dodecyl sulphate (SDS) and the proteins were detected by autoradiography. Up to 24 labelled proteins were detected in the individual cell cultures, but the proteins labelled differed considerably in the 6 cultures examined. A possible reason for this, involving variation in the glycosylation of cell-surface glycoproteins is discussed. Cells of the same melanoma line had similar cell-surface proteins at different passage levels, but changes in the labelled proteins occurred when the culture conditions were altered. The cell-surface proteins of high molecular weight were cleaved by trypsin, but most of the low mol.-wt. proteins were resistant to trypsin. The "large external transformation sensitive" (LETS) protein detected as a major protein on fibroblasts in culture was not a dominant protein on the melanoma cells. It was detected on only 4/6 cell cultures. Possible relationships of the cell-surface proteins described in this study to morphology, immunological properties and proteolytic activity of human melanoma cells are discussed.**

CELLS interact with each other and with their environment by way of their surfaces, and surface structures are thought to be involved in a variety of phenomena such as tumour metastases, escape from immunological attack, growth regulation, and differentiation (Nicolson *et al.*, 1975). An understanding of these phenomena would be facilitated by detailed information on cell surfaces. In an approach to this problem, we have studied the cell-surface proteins of human melanoma cells by a radioiodination technique, followed by separation of the labelled proteins by polyacrylamide-gel electrophoresis and detection of the proteins by autoradiography.

### MATERIALS AND METHODS

*Cells.*—Melanoma and fibroblast cell cultures were developed from operative biopsies and cultured as described by Whitehead (1976). The medium used was McCoy's 5A (Gibco-Biocult Ltd., Paisley) containing 15% foetal calf serum (FCS), 2 mM glutamine, 2 μg/ml insulin, 15 mM HEPES, 1% MEM vitamins, 50 μg/ml penicillin, 50 μg/ml streptomycin and 20 mM $NaHCO_3$. In some experiments the FCS was replaced by 5% human serum.

Two of the melanoma cell cultures (HTCs 163 and 447) were derived from nodular primary tumours and 4 (HTCs 312, 364, 367 and 436) from secondary lymphnode deposits. All the cell cultures were from Caucasians, 2 of whom were males (HTCs 163 and 436). Melanoma cell lines HTC 163 and 312 have been described by Whitehead (1976). HTC 364 contains pigmented, spindle-shaped cells which are aneuploid with a mode of 60. The cells of HTC 367 are spindle shaped and non-pigmented, but melanosomes and premelanosomes were observed on electron-microscopic examination. HTC 436 contains cells which are aneuploid with a mode of 59. The cells of HTC 447, which are pigmented and spindle shaped, have only been used at an early passage level.

*Iodination.*—The method described by

Hubbard and Cohn (1972) was used. Cell monolayers on plastic Petri dishes (5 cm diameter) were washed ×3 with 4 ml phosphate buffered saline (PBS), pH 7·4, and then iodinated with a mixture containing 60 $\mu$Ci of carrier-free $^{125}$I (The Radiochemical Centre, Amersham, Bucks), 2·8 $\mu$mol glucose, 5 u lactoperoxidase (Sigma Chemical Co., Kingston-upon-Thames, Surrey) and 3 u glucose oxidase (Sigma Chemical Co.) in 1 ml PBS for 15 min at room temperature. After removal of the supernatant, the cell monolayer was washed ×3 with 4 ml 0·01 M sodium phosphate buffer, pH 7·4, containing 0·15 M NaI and 2 mM phenylmethyl sulphonyl fluoride (PBI+PMSF) and the cells then scraped from the plate with a rubber policeman and harvested by centrifugation.

The cell pellet was extracted with 0·05 M tris-HCl buffer, pH 6·8, containing 2% SDS, 1% mercaptoethanol, 10% glycerol, 0·001% bromophenol blue and 2 mM PMSF for 3 min at 100°C. In some experiments the mercaptoethanol was omitted.

*Electrophoresis.*—SDS-gel electrophoresis was carried out on a 5–22·5% polyacrylamide gradient gel using the buffer system described by Laemmli (1970) in a slab-gel apparatus constructed as described by Studier (1973). After electrophoresis overnight with a

FIG. 1.—Autoradiograph of $^{125}$I-labelled cells after electrophoresis on 5–22·5% polyacrylamide gels in the presence of SDS. (a) Control plate with no cells; (b) human fibroblast culture; (c) melanoma culture HTC 436; (d) melanoma culture HTC 447; (e) melanoma culture HTC 364.

voltage difference of 40 V across the plate, the gels were stained with 0·05% Coomassie Blue in methanol:water:acetic acid (113:113:23) and destained in methanol:water:acetic acid (50:875:75). The gels were dried on to Whatman No. 3 chromatography paper and subjected to autoradiography using Osray M3 X-ray film.

Electrophoresis under these conditions was shown to be reproducible by electrophoresis of aliquots of the same sample of iodinated cells on gradient gels prepared at different times. Variation in the sample:SDS ratio from half to double the amount normally used, produced identical electrophoretic patterns. The sample was normally applied in a volume of 10 $\mu$l but identical electrophoretic patterns were obtained when the sample volume was varied between 4 and 20 $\mu$l.

Protein standards used for mol. wt. estimation were lysozyme, chymotrypsinogen, bovine serum albumin, phosphorylase a, $\beta$-galactosidase and polymers of lysozyme and bovine serum albumin prepared as described by Payne (1973).

### RESULTS

### *Radiolabelled proteins of melanoma cell cultures*

Autoradiographs of the cell-surface proteins of melanoma cell cultures HTC 364 (passage 26), HTC 436 (passage 11), HTC 447 (passage 2) and an adult fibroblast culture, are shown in Fig. 1. Up to 24 radioactive bands were visible in the individual melanoma cell cultures, but only 8 of these were common to all 6 melanoma cell cultures examined and 7 of these bands were also detected on fibroblast cultures. Estimation of the mol. wts. of these bands by comparison with standards showed that the molecular weights of the cell surface proteins ranged from ~10,000 to 240,000. These values are approximations and should be regarded with some reservation, because many of the radioactive bands are composed of glycoproteins which bind less SDS than non-glycosylated proteins on a w/w basis (Pitt-Rivers and Impiombato, 1968) and consequently for a given mol. wt. will not migrate as rapidly as proteins. The protein which was common to all 6 melanoma cell cultures but which was not found on fibroblasts had a mol. wt. ~140,000.

The LETS protein (approximate mol. wt. 220,000–250,000) described by several workers (Hynes, 1976) was the dominant cell-surface protein in the fibroblast cul-

FIG. 2.—Comparison of the $^{125}$I-labelled proteins from melanoma cell line HTC 163 after culture in (a) 15% FCS and (b) 5% human serum. Proteins were separated on 5–22·5% polyacrylamide gels in the presence of SDS and detected by autoradiography.

tures, but was not a major surface protein in melanoma cells. In 2 of the cell cultures (HTC 312 and 364) the LETS protein was not detected, and in the other 4 cultures it was present but the intensity of the band varied.

FIG. 3.—Autoradiograph of $^{125}$I-labelled melanoma cell line HTC 163 after electrophoresis on 5–22·5% polyacrylamide gels in the presence of SDS. (a) Proteins solubilized with SDS alone; (b) proteins solubilized with SDS and mercaptoethanol; (c) cell pellet extracted with chloroform:methanol (2:1) before solubilization with SDS and mercaptoethanol; (d) chloroform:methanol (2:1) extract from (c).

Incubation of Petri dishes with medium containing FCS for 3 h at 37°C resulted in proteins of low mol. wt. becoming adsorbed on to the plastic surface (Fig. 1). These proteins remained adherent to the plastic after 3 washes with PBS, and required physical disruption and treatment with SDS and mercaptoethanol for solubilization. Proteins with similar migration properties were detected on fibroblasts and melanoma cells, but the amounts of these proteins differed considerably in the different cell cultures. Larger amounts of these proteins were recovered from dishes containing cells than from empty dishes treated with medium and foetal calf serum.

*Effect of passage levels and culture conditions*

The cell-surface proteins of HTC 364 examined at passages 13–16, 19, 22, 24, 26–30, 33 and 35-38 were all similar. Some variations occurred in the relative intensities of the bands, mainly in the low-mol-wt. range, but the general qualitative pattern of cell-surface proteins was stable throughout all the passages. HTC 163 was iodinated at passages 3–5, 8, 13, 14, 20 and 21. Again, the general pattern was similar in all passage levels, although some variations were observed in the intensities of the bands relative to the background, and in the intensities of the low-mol.-wt. proteins relative to each other. In addition, the intensity of the LETS protein varied. Melanoma cell culture HTC 436 was examined at passages 3 and 8–11 and very good agreement was found between the electrophoretic patterns, even the relative intensities of the bands being constant. Cell cultures HTCs 367, 447 and 312 were only examined at passage levels, 14, 2 and 16 respectively.

In contrast to the relative stability of the cell-surface proteins of cells cultured under the same conditions, variation in the culture conditions resulted in greater variation in the cell-surface proteins. A comparison of melanoma cell line HTC 163 grown in FCS with cells grown in human serum is shown in Fig. 2. Although most of the cell-surface proteins in the inter-

FIG. 4.—Effect of digestion with trypsin (10 μg/ml) on the $^{125}I$-labelled proteins from melanoma cell line HTC 364. (a) Untreated; (b) trypsin for 10 min at 20°C; (c) trypsin for 60 min at 20°C. Proteins were separated on 5–22·5% polyacrylamide gels in the presence of SDS, and detected by autoradiography.

mediate size range are similar in cells from both culture conditions, large differences occur in the high- and low-mol.-wt. regions.

*Properties of the cell-surface proteins*

Extraction of radiolabelled melanoma cells with a 2:1 mixture of chloroform and methanol, prior to solubilization with SDS and mercaptoethanol, removed the fast-migrating band which was recovered in the chloroform: methanol extract (Fig. 3). This indicates that the band is probably a lipid. The possibility that this band could be formed by unreacted $^{125}I$ is considered unlikely since a band in this region was not detected when solutions containing free $^{125}I$ were subjected to electrophoresis and autoradiography.

Iodinated melanoma cells extracted with SDS in the presence of mercaptoethanol, and cells extracted with SDS alone, produced electrophoretic patterns which differed in only 2 bands (Fig. 3). These bands were the LETS protein and a band with mol. wt. 55,000, which were present in mercaptoethanol-treated sample but not in the sample solubilized with SDS alone. This result shows that most of the cell-surface proteins of melanoma line HTC 163 are composed of single polypeptide chains rather than multiple chains linked by disulphide bonds. A similar result was obtained with melanoma line HTC 364.

Digestion of radiolabelled line HTC 364 with a dilute trypsin solution (10 μg/ml) prior to solubilization and electrophoresis of the cell pellet removed much of the high-mol.-wt. bands within 10 min at 20°C (Fig. 4). Extension of the digestion period to 1 h at 20°C almost completely removed most of the high-mol.-wt. bands. In contrast to the high-mol.-wt. bands, most of the low-mol.-wt. proteins were resistant to trypsin. One exception to this was a low-mol.-wt. protein which had previously (Fig. 1) been shown to be derived from the culture medium. This protein was completely removed within 10 min by trypsin digestion.

### DISCUSSION

The cell-surface proteins of animal cells such as mouse, hamster and chicken fibroblasts have been the subject of a number of investigations, and changes have been shown in the cell-surface proteins when these cells are transformed by viral or chemical agents (Hynes, 1976). In contrast, apart from the studies of Butters and Hughes (1974, 1975) on KB cells, the cell-surface proteins of human tumour cells have received little attention. In the present study, melanoma cells were chosen because this is one of the few types of human tumour cell which can be consistently cultured *in vitro*. Furthermore, in view of the widespread use of this tumour type in studies on human tumour immunology, a knowledge of the surface proteins of this cell type would facilitate interpretation of immunological observations and identification of the antigens involved.

One of the most important observations in the present study is the large differences in surface proteins of different melanoma cell cultures. The SDS electrophoretic technique used would not be expected to differentiate between genetic variants differing in a few amino-acid substitutions, because this method separates molecules according to size. Most of the differences in the cell-surface proteins from different cultures occurred in regions occupied by molecules which have recently been shown by lectin-binding experiments to be glycoproteins (Roberts, unpublished). Experimentally induced transformation of animal cells is known to lead to changes in the cell-surface glyoconjugates (Buck *et al.*, 1971; Hakomori, 1975; Smets *et al.*, 1975). This raises the interesting possibility that the changes observed in the cell-surface components of different melanoma cultures could be the result of differences in the degree of glycosylation of cell-surface glycoproteins. If this were so, glycoproteins with the same polypeptide core, and conceivably, therefore, the same function in the membrane, but with different molecular sizes, could occur in the different melanoma cell cultures. In view of recent developments implicating the carbohydrate groups of membrane glycoproteins as mediators of cellular recognition phenomena (Ashwell and Morell, 1977) changes in carbohydrate regions of glycoproteins on their surfaces could have important implications in the antisocial behaviour of tumour cells.

Only one labelled band with mol. wt. of ~140,000 was found to be common to all melanoma cells but absent from fibroblasts. It would be premature to claim that this was a melanoma-specific cell-surface component, in view of the small number of melanoma cell cultures examined and the fact that a suitable normal counterpart was not available for comparison in this study. On the other hand, this study does not preclude the presence of other common melanoma antigens, since molecules differing in degree of glycosylation but with the same antigenic activity might migrate to different extents in the electrophoretic system used.

The LETS protein has been the subject of many investigations in the last 4 years, and although the appearance of this protein on normal cells but not on transformed cells (Hynes, 1973) or on cells arrested in mitosis (Hynes and Bye, 1974; Pearlstein and Waterfield, 1974) originally suggested a function for this protein in growth regulation, it is now considered more probable that it is involved in cell adhesion and morphology (Yamada *et al.*, 1976). In the present study there were no apparent differences in the cell morphology of melanoma cells, whether the LETS protein was present or not. The amounts of the LETS protein on the melanoma cells were, however, much less than on fibroblasts.

In addition to protein iodination, a fast-migrating band, soluble in lipid solvents, was also labelled on melanoma and fibroblast cultures. Previous reports (Hubbard and Cohn, 1976) have shown that small amounts of lipids may be labelled during the lactoperoxidase-catalysed iodination method, probably by iodination of the unsaturated bonds in the fatty-acid chains (Butters and Hughes, 1975). The lipid iodination may involve a direct iodination by free iodine which is often present in preparations of iodide nucleides (Morrison, 1974).

The sensitivity of melanoma cell-surface components of high mol. wt. to trypsin-digestion is in agreement with the observations of other workers using hamster fibroblasts (Hynes, 1973), baby hamster kidney fibroblasts (Mastro *et al.*, 1974) and mouse fibroblasts (Hunt and Brown, 1975). This observation indicates that the use of trypsin for removal of melanoma cells from monolayer cultures prior to immunological testing should be avoided. One of the low-mol.-wt proteins was also cleaved by trypsin, but this was a protein derived from the culture medium, and may be extrinsic or peripheral protein according to the Singer and Nicolson classification (1972). It is intriguing that most of the other small proteins are resistant to trypsin. The possibility that these are intracellular proteins is considered unlikely, because the lactoperoxidase-catalysed iodination has now been well tested and shown to label only surface proteins (Hubbard and Cohn, 1972; 1975). Furthermore, the autoradiographic patterns of the labelled cell-surface proteins bore no resemblance to the total cell protein pattern detected by Coomassie-blue staining. One possibility is that these small proteins represent the protein stubs remaining in the membrane after proteolytic digestion of larger cell-surface components. Laico *et al.* (1970) reported the presence of "miniproteins" in membranes from erythrocytes, mitochondria and retinal rods, but these are now thought to have been formed by proteolytic degradation during isolation (Hughes, 1976). In the present investigation, a broad-range protease inhibitor, phenylmethyl sulphonyl fluoride was included in all solutions after iodination. Consequently, proteolytic degradation of the cell-surface proteins is unlikely to have been mediated by proteases released by cellular disruption during isolation. It is considered more probable that proteolysis of the cell-surface proteins occurred during normal culture of the melanoma cells. In support of this possibility there are several reports that transformed cells produce elevated levels of protease, including a cell factor that converts the serum proenzyme plasminogen into the protease plasmin (Bosmann, 1972; Schnebli, 1972; Goldberg, 1974; Unkeless *et al.*, 1973). Recently it has been shown that human melanoma cells possess a surface protease (Hatcher *et al.*, 1976).

I wish to thank Miss Gwenda M. Roberts and Dr R. H. Whitehead for supplying the cell cultures used in this investigation and Professor L. E. Hughes for advice. This study was supported by a grant from the Cancer Research Campaign.

### REFERENCES

Ashwell, G. & Morell, A. G. (1977) Membrane glycoproteins and recognition phenomena. *Trends. Biochem. Sci.*, **1**, 76.

Bosmann, H. B. (1972) Elevated glycosidase and proteolytic enzymes in cells transformed by RNA tumor virus. *Biochem. Biophys. Acta*, **264**, 339.

Buck, C. A., Glick, M. C. & Warren, L. (1971) Glycopeptides from the surface of control and virus-transformed cells. *Science*, **172**, 169.

Butters, T. D. & Hughes, R. C. (1974) Solubilization and fractionation of glycoproteins and glycolipids of KB cell membranes. *Biochem. J.*, **140**, 469.

Butters, T. D. & Hughes, R. C. (1975) Surface labelling of human tumour KB cells. Iodination and fractionation of membrane glycoproteins. *Biochem. J.*, **150**, 59.

Goldberg, A. R. (1974) Increased protease levels in transformed cells: a casein overlay assay for the detection of plasminogen activator production. *Cell*, **2**, 95.

Hakomori, S. (1975) Structure and organization of cell surface glycolipids: dependency on cell growth and malignant transformation. *Biochem. Biophys. Acta*, **417**, 55.

Hatcher, V. B., Wertheim, M. S., Rhee, C. Y., Tsien, G. & Burk, P. G. (1976) Relationship between cell surface protease activity and doubling time in various normal and transformed cells *Biochem. Biophys. Acta*, **451**, 499.

Hubbard, A. L. & Cohn, Z. A. (1972) The enzymatic iodination of the red cell membrane. *J. Cell Biol.*, **55**, 390.

Hubbard, A. L. & Cohn, Z. A. (1975) Externally disposed plasma membrane proteins. 1. Enzymatic iodination of mouse L cells. *J. Cell Biol.*, **64**, 438.

Hubbard, A. L. & Cohn, Z. A. (1976) Specific labels for cell surfaces. In *Biochemical Analysis of Membranes* Ed. A. D. Maddy. London: Chapman and Hall. p. 427.

Hughes, R. C. (1976) *Membrane Glycoproteins. A Review of Structure and Function*. London: Butterworth's.

Hunt, R. C. & Brown, J. C. (1975) Identification of a high molecular weight transmembrane protein in mouse L cells. *J. Molec. Biol.*, **97**, 413.

Hynes, R. O. (1973) Alterations of cell-surface proteins by viral transformation and by proteolysis. *Proc. Natl. Acad. Sci. U.S.A.*, **70**, 3170.

Hynes, R. O. (1976) Cell surface proteins and malignant transformation. *Biochem. Biophys. Acta*, **458**, 73.

Hynes, R. O. & Bye, J. M. (1974) Density and cell cycle dependence of cell surface proteins in hamster fibroblasts. *Cell*, **3**, 113.

Laemmli, U. K. (1970) Cleavage of structural proteins during the assembly of the head of bacteriophage T4. *Nature*, **227**, 680.

LAICO, M. T., RUOSLAHTI, E. I., PAPERMASTER, D. S. & DREYER, W. J. (1970) Isolation of the fundamental polypeptide subunits of biological membranes. *Proc. Natl. Acad. Sci., U.S.A.*, **67**, 120.

MASTRO, A. M., BEER, C. T. & MUELLER, G. C. (1974) Iodination of plasma membrane proteins of BHK cells in different growth states. *Biochem. Biophys. Acta*, **352**, 38.

MORRISON, M. (1974) The determination of the exposed proteins on membranes by the use of lactoperoxidase. In *Methods in Enzymology Vol. 32. Biomembranes Part B.* Eds. S. Fleischer and L. Packer. London: Academic Press. p. 103.

NICOLSON, G. L., ROBBINS, J. C. & WINKELHAKE, J. L. (1975) Tumor cell surfaces and metastasis: dynamic changes in neoplastic membrane structure and their relationship to tumor spread. In *Cellular Membranes and Tumour Cell Behaviour.* Baltimore: Williams and Wilkins. p. 81.

PAYNE, J. W. (1973) Polymerization of proteins with glutaraldehyde. Soluble molecular-weight markers. *Biochem. J.*, **135**, 867.

PEARLSTEIN, E. & WATERFIELD, M. D. (1974) Metabolic studies on $^{125}$I-labelled baby hamster kidney cell plasma membranes. *Biochem. Biophys. Acta*, **362**, 1.

PITT-RIVERS, R. & IMPIOMBATO, F. S. A. (1968) The binding of sodium dodecyl sulphate to various proteins. *Biochem. J.*, **109**, 825.

SCHNEBLI, H. P. (1972) A protease-like activity associated with malignant cells. *Schweiz. Med. Wochenschr*, **102**, 1194.

SINGER, S. J. & NICOLSON, G. L. (1972) The fluid mosaic model of the structure of cell membranes. *Science*, **175**, 720.

SMETS, L. A., VAN BEEK, W. P., COLLARD, J. G., TEMMINK, H., VAN GILS, B. & EMMELOT, P. (1975) Comparative evaluation of plasma membrane alterations associated with neoplasia. In *Cellular Membranes and Tumor Cell Behaviour*. Baltimore: Williams and Wilkins. p. 269.

STUDIER, F. W. (1973) Analysis of bacteriophage T7 early RNAs and proteins on slab gels. *J. Molec. Biol.*, **79**, 237.

UNKELESS, J., TOBIA, A., OSSOWSKI, L., QUIGLEY, J. P., RIFKIN, D. B. & REICH, E. (1973) An enzymatic function associated with transformation of fibroblasts by oncogenic viruses. I. Chick embryo fibroblast cultures transformed by avian RNA tumor viruses. *J. Exp. Med.*, **137**, 85.

WHITEHEAD, R. H. (1976) The culture of tumour cells from human tumour biopsies. *Clin. Oncol.*, **2**, 131.

YAMADA, K. M., YAMADA, S. & PASTAN, I. (1976) Cell surface protein partially restores morphology, adhesiveness and contact inhibition of movement to transformed fibroblasts. *Proc. Natl. Acad. Sci., U.S.A.*, **73**, 1217.